# Nuptial gifts fail to resolve a sexual conflict in an insect

**DOI:** 10.1186/1471-2148-8-204

**Published:** 2008-07-15

**Authors:** Nina Wedell, Tom Tregenza, Leigh W Simmons

**Affiliations:** 1 Centre for Ecology and Conservation, School of Biosciences, University of Exeter, Cornwall Campus, Penryn, TR10 9EZ, UK; 2 Centre for Evolutionary Biology, School of Animal Biology (M092), The University of Western Australia, Crawley, WA 6009, Australia

## Abstract

**Background:**

Because of the potential benefits to individuals of saving investment for future mating opportunities, there is conflict between mates over most aspects of reproduction. Males of many species transfer compounds in the ejaculate that manipulate female reproductive physiology to increase male reproductive success. These seminal compounds are often associated with direct and/or indirect costs to females. In contrast, in some species ejaculates also contain nutrients used by females for somatic maintenance and increased reproductive output. In general, the extent to which male seminal components are detrimental or beneficial to females is poorly understood, and interactions between seminal compounds with different effects have been almost completely neglected. Here we examine the impact of male receptivity-suppressing factors and nutrient donations on female longevity and lifetime reproductive output in the bushcricket *Requena verticalis*.

**Results:**

We show that receiving multiple ejaculates reduces longevity in female *R. verticalis*, indicating a cost of male derived receptivity-suppressing compounds. Consumption of male nutrient donations does not appear to ameliorate this longevity cost, and there was no effect of nutrient provisioning on female lifetime fecundity.

**Conclusion:**

These results indicate that nutrient provisioning does not provide a resolution to sexual conflict over female receptivity in this bushcricket species.

## Background

The reproductive interests of mates rarely coincide, resulting in sexual conflict over most aspects of reproduction [1,2]. There is frequent conflict over female remating, as males will have to endure sperm competition and reduced fertilization success when females mate again [3]. The risk of sperm competition has promoted a variety of male adaptations, often associated with female costs [4]. Males frequently transfer compounds in the ejaculate that manipulate female reproductive physiology to increase male reproductive success. For example, *Drosophila melanogaster *males transfer a cocktail of >80 different proteins in the ejaculate that (amongst other things) stimulate oviposition and reduce female receptivity, thereby increasing male fertilization success [5,6]. However, these male-derived molecules have a negative effect on female fitness by reducing lifespan [7]. It has even been suggested that male harm could evolve as a means to manipulate females to increase their terminal investment in immediate reproductive output, due to reduced residual reproductive value, which then results in higher male reproductive success [8,9].

Not all compounds transferred to the female at mating have a negative effect on female fitness. Males of several insects transfer nutrients at mating, either in the ejaculate or together with the sperm packet, that increase female reproductive success by enhancing fecundity and/or offspring survival [10-13]. As a consequence, male nutrient donations create an additional conflict over mating; a female should remate and obtain additional nutrients to increase her fecundity, whereas a male should prevent the female from remating to ensure paternity of the offspring in which he invests [14]. For example, in *Pieris napi *butterflies, male spermatophores contain nutrients increasing both female fecundity and longevity [15], and anti-aphrodisiacs that render females unattractive to rival males following mating [16], as well as large numbers of non-fertile sperm that switch off long-term female receptivity [17]. Nutrient donations can represent a substantial investment by male insects, at times approaching or even exceeding that of female investment in egg production [18,19], and require extended periods of male recouperation [20,21]. It is therefore likely that the greater the value to females of receiving male donations together with the costs to males of providing such gift, will exacerbate the level of sexual conflict over female remating rate.

In species where the cost of providing nutrients directly limits male mating rate, there is particularly strong selection on males to reduce female remating and the ensuing sperm competition, and opposing selection on females to increase their mating rate. Male insects have evolved a variety of ways to reduce female remating, ranging from mate guarding and physical barriers preventing additional copulations, to transfer of various receptivity suppressing ejaculate components, including large numbers of non-fertile sperm [5,22-24]. These adaptations are known to be effective in reducing female remating rates, even if not successfully preventing female remating altogether [4]. Some of these male adaptations are known to impose costs on females [4], which may also be borne by the manipulating male, if production of his offspring is reduced. This creates a somewhat paradoxical situation in which males compromise their own fitness by harming their mates, a paradox which is normally explained by the potential for the benefits to males from reduced female remating to exceed the costs from reduced fecundity. However, if males reduce female fecundity below its maximum, this creates selection for a male adaptation that restores female fecundity, which provides a resolution to this paradox.

Males of many species of bushcrickets (Orthoptera: Tettigoniidae) provide females with a large nuptial gift, synthesized by the male at mating, that increase female fecundity and offspring fitness [10,13,25]. Nutrient provisioning is often associated with male mating costs in terms of time required before being able to produce a new spermatophore and mate again [20,21,26]. The spermatophore of the univoltine Australian bushcricket *Requena verticalis *is, like most bushcricket species, comprised of a sperm-containing ampulla and a sperm-free gelatinous spermatophylax that contains male-derived nutrients that can directly enhance female fecundity [25]. The entire spermatophore is attached externally to the females' genital opening at mating. The female removes and feeds on the sperm-free spermatophylax during sperm transfer from the ampulla, and will later remove and consume the empty ampulla. The spermatophylax in *R. verticalis *therefore serves a dual role of both protecting the ejaculate during insemination, and as a paternal investment [27]. Males incur a substantial cost of spermatophore production requiring several days before being able to mate again [28]. Following mating the female enters into a non-receptive period, which is directly related to the amount of ejaculate transferred [29]. However, the duration of a female's non-receptivity period depends on her nutritional status [30], suggesting substantial sexual conflict over female remating rate [31].

Previous work has shown nutrients provided in the spermatophylax increase immediate female fecundity, egg weight and offspring survival [25,32]. However, the impact of spermatophylax consumption and amount of ejaculate received on female lifetime fecundity or longevity has not been examined. This is despite the frequently observed negative impact on female lifespan of receptivity-suppressing compounds transferred in the ejaculate of several insect taxa [1]. To examine the hypothesis that potentially costly manipulative ejaculates can be compensated for by simultaneous provisioning of nutrients, we examine the impact of varying the amount of ejaculate received, and spermatophylax consumption on female lifespan and lifetime reproductive output. We specifically ask whether male spermatophylax provisioning can compensate for the potential cost to females of receiving manipulative ejaculates, which may indicate that males provide nutrient to restore female fecundity, whilst simultaneously enjoying high paternity by reducing the risk of sperm competition. We show that ejaculate receipt is costly to females in terms of reduced longevity, and that spermatophylax consumption does not appear to ameliorate this cost.

## Results

**Figure 1:**
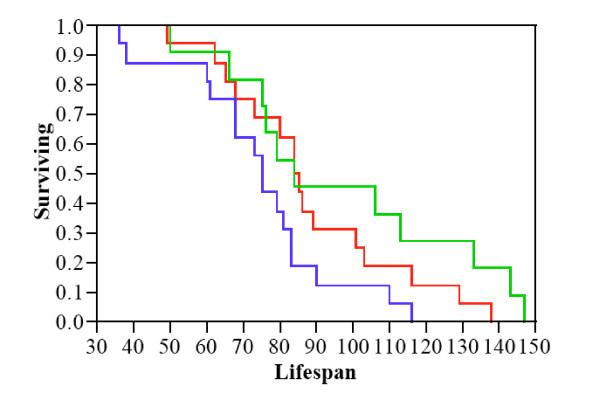
**Female longevity in relation to spermatophylax consumption and number of ejaculates received**. Females that receive three ejaculates (blue line) die sooner than females receiving only one ejaculate (green line). Spermatophylax consumption does not affect female lifespan (red line).

Female lifespan ranged between 36 – 147 days post first copulation, and differed with respect to mating treatment. There was a significant effect of our mating treatments on female lifespan (χ^2 ^= 6.59, p = 0.037, Fig 1), but no effect of either female size (χ^2 ^= 1.49, p > 0.2) or lifetime fecundity (χ^2 ^= 0.47, p > 0.4). Planned comparison of the impact of the amount of ejaculate received on longevity revealed a cost of ejaculate receipt, with females receiving three full ejaculates dying sooner than females receiving one ejaculate (χ^2 ^= 4.54, p = 0.033), and no effect of either female size (χ^2 ^= 0.083, p > 0.7) or lifetime fecundity (χ^2 ^= 0.15, p > 0.6) on lifespan. However, the planned comparison examining the impact of spermatophylax consumption showed there was no difference in longevity between females receiving one ejaculate with respect to consuming spermatophylax material or not (χ^2 ^= 1.29, p > 0.2), with again no influence of female size (χ^2 ^= 0.74, p > 0.3) or lifetime fecundity (χ^2 ^= 0.001, p > 0.9). On average, females receiving three ejaculates lived 75 days compared to 84 and 84.5 days for females receiving one ejaculate only, or one ejaculate and allowed to consume three spermatophylaces, respectively. The cost in terms of reduced lifespan of receiving three ejaculates versus one is ~11 days, whereas the (non-significant) positive effect of consuming three spermatophylaces versus none (when receiving one ejaculate) is only ~ half a day. This clearly indicates a cost to females of receiving male ejaculates in terms of reduced longevity, and no impact on lifespan of spermatophylax consumption. We therefore conclude that spermatophylax consumption do not ameliorate the cost of receiving multiple ejaculates in *R. verticalis*.

**Figure 2:**
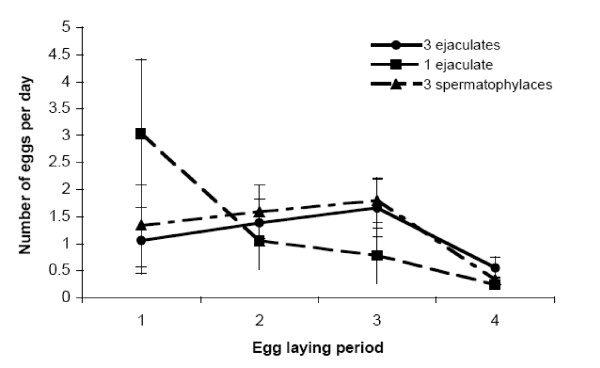
**Egglaying rate in relation to mating treatment**. There is no difference in female egg-laying rate with respect to spermatophylax consumption or number of ejaculates received in either of the 4 egg-laying periods, and no influence of female size (p > 0.14 – > 0.99).

To clarify that any potential fecundity effects were not confounded by differences in longevity between treatments, we examined the impact of mating treatment across all females. There was no difference between mating treatments with respect to female lifetime fecundity, mean egg weight, the total mass of eggs produced, or egg-laying rate (Table 1, Fig 2). Although bigger females had higher total reproductive output (total mass of eggs laid: F_1,41 _= 4.68, p = 0.037) they did not lay heavier eggs (F_1,41 _= 2.69, p > 0.11). Across all females there was no relationship between female longevity and fecundity (F_1,41 _= 0.30, p > 0.58), mean egg weight (F_1,41 _= 0.004, p > 0.95), or between longevity and female size (F_1,41 _= 0.51, p > 0.48). Mating treatment did also not affect the onset of egg-laying following mating (Likelihood ratio χ^2 ^= 9.34, p > 0.16).

**Table 1:** The effect of mating treatment on female reproduction

		Mating treatment			
Variable	***F***_2,41_	1 Ejaculate	3 Ejaculates	Spermatophylax	*p*
Lifetime fecundity	0.167	41.9 ± 9.8	42.3 ± 7.4	47.4 ± 8.1	>0.8
Mean egg weight	1.089	2.29 ± 0.06	2.15 ± 0.05	2.18 ± 0.06	>0.3
Mass of eggs laid	0.214	92.4 ± 19.1	89.2 ± 15.8	103.5 ± 15.8	>0.8
Oviposition rate 0–5 days	1.009	3.04 ± 1.38	1.06 ± 0.61	1.34 ± 0.76	>0.3
Oviposition rate 6–10 days	0.328	1.05 ± 0.53	1.39 ± 0.44	1.59 ± 0.49	>0.7
Oviposition rate 11–15 days	1.253	0.78 ± 0.50	1.66 ± 0.52	1.80 ± 0.41	>0.2
Oviposition rate ≥ 16 days	1.833	0.24 ± 0.04	0.55 ± 0.20	0.33 ± 0.05	>0.1

The effect of mating treatment on female lifetime fecundity, mean egg weight (mg), mass of eggs laid (mg), and oviposition rate (eggs per day) in either of the four egg laying periods, together with means ± SE for each variable per treatment.

## Discussion

Our results show that receiving multiple copulations and/or receiving multiple ejaculates reduces female *R. verticalis *lifespan, and that consumption of male nutrient donations cannot ameliorate this cost. Our experimental design cannot separate possible effects on female lifespan of multiple copulations from those of ejaculate compounds. However, matings do not appear to involve any behaviour that might directly impose costs on females, at least in the laboratory where predation is absent. Males attach the spermatophore externally to the females' genital opening; hence insemination occurs unaided by any male intromittent organ. Previous work in *R. verticalis *demonstrates that the ejaculate contains factors that suppress female receptivity, and that this effect is directly related to the amount of ejaculate received [29]. Similar findings come from studies of a number of other bushcricket species, indicating that the ejaculate in general contains receptivity-suppressing compounds in this insect family [33-35]. In general, receptivity-suppressing compounds are transferred to the female at mating in several insects [e.g. [7,22,24,36]]. It is not clear what these compounds are, but in gryllid crickets prostaglandins or prostaglandin precursors are transferred in the seminal fluid to females where they trigger oviposition [33,37-40]. Male gryllid crickets also transfer > 30 different additional seminal proteins, which show evidence of rapid evolution indicative of ongoing selection, although their function in regulating female reproduction is, as yet, unclear [41]. In contrast to the marked effects of ejaculates, there is no evidence that spermatophylax consumption has any effect on female *R. verticalis *receptivity [29].

Previous work has shown that female *R. verticalis *incorporate male derived nutrients passed in the spermatophylax into their soma, and that they retain a larger amount of male nutrients when experiencing nutritional stress [42]. However, our results found no evidence that females make use of nutrients in the spermatophylax for their somatic maintenance to thereby ameliorate the longevity cost of receiving ejaculates. This suggests that male derived nutrients are not important to female fitness in terms of increasing lifespan. The importance of male derived nutrients to female fitness has previously been demonstrated in the bushcricket *Kawanaphila nartee*, where the duration of the female's refractory period is dependent not only on the amount of ejaculate received, but also on their diet, with females in greater nutritional need having shorter periods of non-receptivity [31]. Females appear to overcome male manipulation when the value of males' nutrient donations is large. The same may be true for *R. verticalis*, since poorly nourished females have a shorter refractory period than well-fed females [30], although it is not clear from our results that this carries any direct benefits in terms of increased longevity or lifetime fecundity. It is possible that the low protein diet females were kept on reduced their ability overall to produce eggs relative to females in the field, where presumably they encounter a more varied diet and hence a longer lifespan may translate to increased egg production.

In other insects, females do make use of male derived nutrients to extend their lifespan [36]. For example, in the green-veined white butterfly (*P. napi*), male derived nutrients are important to female fitness not only by increasing fecundity, but also by allowing old females to histolyse their wing muscles and convert these resources into eggs [43]. Male nutrient provisioning therefore ensures females live sufficiently long to have time to convert their wing muscles into more eggs. Similarly, in the comma butterfly (*Polygonia c-album*), male-derived nutrients allow females to increase their reproductive output without associated longevity costs since they can make use of male donations for their somatic maintenance [20]. In some gryllid crickets, female multiple mating is also associated with enhanced longevity, although it is not clear if this is directly due to benefits from spermatophore consumption, as the effect was observed in species lacking a nutritious spermatophylax [44]. Similarly, in the cricket *Gryllus lineaticeps*, males do not provide a spermatophylax, yet females mating multiply to preferred males enjoy both increased longevity and fertility, which is likely due to variation in seminal fluid quality between males [45].

Contrary to previous work [e.g. [25,32]], we did not find that females allowed to consume three spermatophylaces laid more or heavier eggs in their lifetime than females prevented from spermatophylax consumption. To examine whether the lack of observed difference in female reproductive output could be explained by low power in our experiment relative to previous published results [e.g. [25]], we examined the effect sizes of spermatophylax feeding on egg weight as this measure is comparable in the two studies [46,47]. The sample sizes of the experiments are of similar magnitude (12–14 versus 11–16, our study), and the effect size in our study was roughly half (*r *= 0.142) that of Gwynne's [25] (*r *= 0.316). Overall, eggs were heavier in our study compared to those in Gwynne [25] (range 2.15 – 2.29 versus 1.94 – 2.06 mg), but were similar in weight to that reported by Gwynne and co-workers [48] (2.15 – 2.19 mg), indicating substantial variation among females in this trait. Previous work has shown that adult diet can directly influence female fecundity, but not egg weight [32,42], so that the effect of spermatophylax consumption on female fecundity may depend on adult female diet. One possible explanation for the difference in the effect of spermatophylax consumption between the studies therefore, is that we did not restrict the diet sufficiently for the females in our study. We think this unlikely as we used the same low quality adult diet, composed solely of rolled oats and water, as that used by Gwynne [32], who also showed that the effect of spermatophylax consumption on fecundity was not dependent upon adult diet manipulation. Although we cannot rule out there may be subtle differences in the nutrient composition of the diet between the experiments that may affect female fecundity, this cannot explain the absence of an effect of spermatophylax consumption in out study. Additionally, here we measured female lifetime fecundity and egg production using a mating frequency characteristic of natural populations [49], whereas previous work examined the effect of spermatophylax consumption on female fecundity and egg weight over a much shorter time scale (~first 30 days after mating). A further reason for the differences may be that Gwynne [32] used shorter remating intervals than females experienced in this study, which were chosen to reflect the mating frequency observed in natural populations [49]. It would thus appear that the main function of the spermatophylax in this species is to protect the ejaculate during insemination, but that it may also serve to provide nutrients to females during some circumstances [27].

It is not clear why females of some species are able to retain some control over their reproduction whereas other species seem to be at the mercy of manipulative males. Sexual conflict generates continuous adaptation and counter-adaptation of reproductive traits by the sexes [50-52], which can lead to rapid elaboration of traits important to reproductive success [1]. For example, there is evidence that male seminal products are under strong positive selection, and are rapidly evolving in both insects and vertebrates, including man [53-55]. The reason for the rapid evolution of seminal traits is thought to be that males are selected to continuously evolve more potent compounds, as females evolve increased resistance to male manipulation [50]. At any point in time, either males or females may have the 'upper hand', which may in part explain the observed differences between species in the extent of female control. In addition, the value and cost of female remating varies between species, which could influence the outcome. It has been proposed that females may gain the upper hand in sexual conflict when the value of 'winning' is greater and the cost of resisting manipulative males is low [56]. Our results suggest that the longevity cost to *R. verticalis *females associated with male induced non-receptivity does not compromise overall female fitness, because it does not appear to affect lifetime fecundity. It is unlikely that females could benefit from reduced lifespan via a reduction in generation time, as *R. verticalis *is univoltine, having an obligate overwinter diapause. The benefits to males of inducing non-receptivity periods may thus outweigh the longevity costs to females, so that manipulative ejaculates can be maintained.

## Conclusion

We demonstrate that male nutrient donations do not ameliorate the longevity costs to *R. verticalis *females of receiving ejaculates with receptivity-suppressing compounds, or provide a direct benefit by increasing their lifetime egg production. Nutrient provisioning does not appear to provide a resolution to sexual conflict over female receptivity in this bushcricket. Our results suggest that substantial conflicts of interest over mating rate can persist, and that matings can continue to be harmful to females even when they involve substantial investment by males.

## Methods

Female *R. verticalis *were collected as last instar nymphs from around the campus of the University of Western Australia. Males were caught either as last instar nymphs or adults. Insects were kept in individual Perspex vials, 6.5 cm in diameter and 15 cm tall. Females were kept on a low protein diet consisting of rolled oats and water. This diet was chosen to maximize the effect of our mating treatment, and should reflect situations of low protein availability in the field. Males were also fed dried cat food as a protein source to ensure they were capable of producing a large spermatophore and associated nutritious spermatophylax [22]. Adult males were kept in the lab in this way for at least a week before being used as mating partners for our experimental females. Experimental females were at least 5 days post adult eclosion before being offered a male for their first copulation (range 5–9 days). Each female was placed into the vial of a singing male. If the female did not show signs of receptivity within 3 hours or attempted to cannibalise the male, she was removed from the vial and presented with a new male the following day until she mated for the first time.

Females were allocated randomly to one of three mating treatments. Females in the first treatment received three ejaculates but no spermatophylax meals. Thus, females were allowed to mate on three occasions separated by 6–10 days. This remating interval was chosen to conform to the natural lifetime mating frequency of field-collected females [49]. Following mating the spermatophylax was removed from the ampulla of the spermatophore with forceps, while ensuring the ampulla remained attached to the female's genital opening. The female was then placed in a narrow tube to prevent her from removing the ampulla. Females were kept in the tube for 190 min following ampulla attachment to ensure complete transfer of the ejaculate [27]. Females in the second treatment received a single ejaculate at their first mating and were also prevented from consuming their spermatophylax. They received no further matings. Females in the third treatment also received a single mating, but they were allowed to consume their spermatophylax. These females received two additional spermatophylaces at 6–10 day intervals. Thus, females received either (1) three ejaculates no spermatophylax; (2) one ejaculate no spermatophylax; or (3) one ejaculate and three spermatophylaces. The females in treatments 2 and 3 were subjected to 190 min sessions in the narrow tube to control for any potential effect that tube exposure may have had on females in treatment 1. All matings were conducted under red light at 28°C.

Following the mating treatment, females were returned to their individual vials, and provided with damp sand as egg laying substrate. The sand was sieved on three occasions at 5 day intervals. The first egg counting interval included the eggs laid by the female in the first 5 days since completion of their mating treatment, and any eggs laid during the time of the mating treatment. Eggs were counted, and weighed to the nearest 0.01 mg. Females were then monitored daily until they died, and all the eggs laid since the last egg check were counted and weighed as before. Lifespan was measured as the number of days since their first copulation until death. Females that laid fewer than 10 eggs during the their lifespan (N = 6) were excluded from the analyses. At death the pronotum width was measured as an estimate of female body size. Overall, there was no difference in female body size with respect to mating treatment (F = 0.15, p > 0.8, N = 43).

### Statistics

The effect of mating treatment on female longevity was examined using Cox's proportional hazards analyses on uncensored data, as we have known lifespan for all females, with female size and lifetime fecundity as covariates. We conducted two planned contrasts. The effect of mating on longevity was examined by contrasting the longevity of females receiving 1 versus 3 ejaculates. The effect of spermatophylax feeding on longevity was determined by contrasting the longevity of females that received a single ejaculate without spermatophylax feeding or given access to three spermatophylaces.

The effect of mating treatment on female reproductive output was examined by looking for differences in lifetime fecundity, the number of eggs laid in each of the four egg counting periods (i.e. 5, 10, 15 days or after mating or between day 16 and until death), egg weight (including egg weight in each of the four egg counting periods), and egg laying rate in each of the four periods. We used Generalized Linear Models specifying a Poisson error distribution (data corrected for over-dispersion) with female pronotum size as a covariate where appropriate. The effect of mating treatment on onset of egg laying was analysed using contingency test. Data analyses were performed in R version 2.2.1 [57].

## Authors' contributions

NW, TT, and LWS contributed to the conceptual development of the work, the experimental work and the writing of this manuscript. NW performed data analyses.
